# Endoscopic Features of Post–COVID-19 Cholangiopathy and Its Management Using ERCP

**DOI:** 10.14309/ajg.0000000000002562

**Published:** 2023-10-16

**Authors:** Silke Leonhardt, Donata Grajecki, Dominik Geisel, Uli Fehrenbach, Andreas Adler, Julia Leonhardt, David Horst, Florian Kurth, Charlotte Thibeault, Hans-Joachim Janssen, Thomas Kaul, Siegbert Faiss, Frank Tacke, Christian Jürgensen

**Affiliations:** 1 Department of Hepatology and Gastroenterology, Charité-Universitätsmedizin Berlin, Corporate Member of Freie Universität Berlin and Humboldt-Universität zu Berlin, Berlin, Germany;; 2 Department of Radiology, Charité-Universitätsmedizin Berlin, Corporate Member of Freie Universität Berlin and Humboldt-Universität zu Berlin, Berlin, Germany;; 3 Department of Anesthesiology and Intensive Care Medicine, University Hospital Jena and Center for Sepsis Control and Care, University Hospital Jena, Jena, Germany;; 4 Institute of Pathology, Charité-Universitätsmedizin Berlin, Corporate Member of Freie Universität Berlin and Humboldt-Universität zu Berlin, Berlin, Germany;; 5 Department of Infectious Diseases and Respiratory Medicine, Charité-Universitätsmedizin Berlin, Corporate Member of Freie Universität Berlin, Humboldt-Universität zu Berlin and Berlin Institute of Health, Berlin, Germany;; 6 Department of Anesthesiology, Intensive Care and Pain Medicine, BG Klinikum Unfallkrankenhaus Berlin gGmbH, Berlin, Germany;; 7 Department of Internal Medicine, BG Klinikum Unfallkrankenhaus Berlin gGmbH, Berlin, Germany.

**Keywords:** secondary sclerosing cholangitis, SSC-CIP, SC-CIP, COVID-19 cholangiopathy, liver abscess, peribiliary liver abscess, biliary cast syndrome, ketamine-induced cholangiopathy, vanishing bile ducts, cholestasis

## Abstract

**INTRODUCTION:**

Despite growing awareness of post–coronavirus disease 2019 (COVID-19) cholangiopathy as one of the most serious long-term gastrointestinal consequences of COVID-19, the endoscopic features of this disease are still poorly characterized. This study aimed to more precisely define its endoscopic features and to outline the role of endoscopic retrograde cholangiopancreatography (ERCP) in the management of this entity.

**METHODS:**

In this observational study, 46 patients with confirmed post–COVID-19 cholangiopathy were included.

**RESULTS:**

Based on the endoscopic features observed in 141 ERCP procedures, post–COVID-19 cholangiopathy can be classified as a variant of secondary sclerosing cholangitis in critically ill patients. It appeared early in the course of intensive care treatment of patients with COVID-19 (cholestasis onset 4.5 days after intubation, median). This form of cholangiopathy was more destructive than stricturing in nature and caused irreversible damage to the bile ducts. A centripetal pattern of intrahepatic bile duct destruction, the phenomenon of vanishing bile ducts, the absence of extrahepatic involvement, and the presence of intraductal biliary casts (85% of patients) were typical cholangiographic features of post–COVID-19 cholangiopathy. This cholangiopathy was often complicated by small peribiliary liver abscesses with isolation of *Enterococcus faecium* and *Candida* spp. in bile culture. The prognosis was dismal, with a 1-year liver transplantation–free survival rate of 44%. In particular, patients with peribiliary liver abscesses or destruction of the central bile ducts tended to have a poor prognosis (n.s.). As shown by multivariate analysis, bilirubin levels (on intensive care unit day 25–36) negatively correlated with liver transplantation–free survival (hazard ratio 1.08, *P* < 0.001). Interventional endoscopy with cast removal had a positive effect on cholestasis parameters (gamma-glutamyl transpeptidase, alkaline phosphatase, and bilirubin); approximately 60% of all individual values decreased.

**DISCUSSION:**

Gastrointestinal endoscopy makes an important contribution to the management of post–COVID-19 cholangiopathy. ERCP is not only of great diagnostic and prognostic value but also has therapeutic value and therefore remains indispensable.

## Graphical abstract

**Figure:**
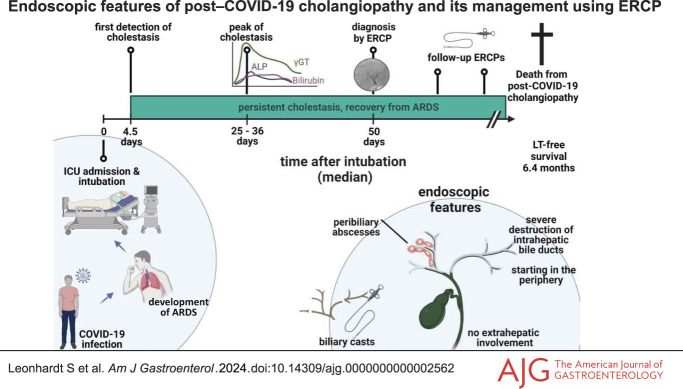

## INTRODUCTION

Several reports of “coronavirus disease 2019 (COVID-19)–associated cholangiopathy” have attracted attention in the past 3 years (1–8). Initially believed to be a new entity, it was suspected that this cholangiopathy was attributable to viral tropism of SARS-CoV-2 to cholangiocytes (2). Other authors postulated that this cholangiopathy observed in patients with COVID-19 was ketamine induced (7). Exclusively observed in critically ill patients, increasing evidence suggests that post–COVID-19 cholangiopathy is (merely) a variant of secondary sclerosing cholangitis in critically ill patients (SSC-CIP) (9,10). As recently shown, the incidence of this post–COVID-19 cholangiopathy is high—up to 2.3 cases per 100 invasively ventilated patients with COVID-19 (9). Accordingly, a high prevalence of patients with post–COVID-19 cholangiopathy can be expected in the post–COVID-19 era. Adequate diagnosis and management of this COVID-19 sequelae is a multidisciplinary challenge. Endoscopic retrograde cholangiopancreatography (ERCP) is currently the gold standard for the diagnosis of SSC-CIP (11). Therefore, the aim of this study was to describe typical endoscopic features of post–COVID-19 cholangiopathy.

## PATIENTS AND METHODS

### Patients

This study analyzed endoscopic findings and clinical data of 46 patients with post–COVID-19 cholangiopathy. All patients included in the analysis developed new-onset cholangiopathy during the course of SARS-CoV-2 infection (confirmed by quantitative reverse transcription polymerase chain reaction). None of the patients had a history of preexisting biliary tract disease. The diagnosis of post–COVID-19 cholangiopathy was established by a characteristic clinical course with progressive cholestasis in conjunction with bile duct lesions in ERCP. The primary recruitment of patients with post–COVID-19 cholangiopathy at the Charité Berlin, Germany, was part of a subproject of the prospective, observational PA-COVID-19 study (trial on the pathophysiology and clinical course of COVID-19, registered in the German clinical trials register on May 13, 2020, ID: DRKS00021688) (12). Thirty-nine patients with COVID-19 hospitalized at Charité University Medical Center Berlin (Charité-Universitätsmedizin Berlin) from March 1, 2020, to April 30, 2022, were considered for inclusion in this study. The study was approved by the local ethics committee of Charité (EA2/066/20 and EA/129/20) and was conducted in accordance with the Declaration of Helsinki and Good Clinical Practice principles (ICH 1996). An additional cohort of 7 patients with post–COVID-19 cholangiopathy from Unfallkrankenhaus Berlin was retrospectively included in the analysis after anonymization of their data. All patients were consecutively enrolled in the study once the diagnosis of post–COVID-19 cholangiopathy was confirmed. All patients were followed up from the time of diagnosis until liver transplantation (LT) or death, the primary end points. The cutoff date for follow-up was April 30, 2022 (follow-up min–max: 24.0–732 days).

### Interventions

ERCP was performed when medically indicated, e.g., for investigation of cholestasis of unclear etiology or suspicion of post–COVID-19 cholangiopathy. All ERCP procedures were performed by experienced endoscopists, and Charité Berlin is a high-volume center for ERCP (>1,500 ERCP/yr). At the end of the observation period, the ERCP findings were reevaluated by 2 investigators (S.L. and C.J.). ERCP was conducted using a standard side-viewing therapeutic duodenoscope (TJF-140F; Olympus America, Melville, NY). The procedure was generally performed under sedation with propofol, but intravenous anesthesia was used in some ventilated intensive care unit (ICU) patients. All patients referred for ERCP had a normal duodenal anatomy with naive papilla and no prior sphincterotomy. Biliary sphincterotomy was performed in all 46 cases either as part of the anticipated endoscopic treatment (cast extraction) or as a protective measure for subsequent ERCP. Procedure-related complications, including bleeding, perforation, and pancreatitis, were recorded. The diagnostic criteria for post-ERCP pancreatitis were defined according to the 1991 consensus report by Cotton et al (13). Clinically significant biliary hemorrhage was defined as clinical evidence of bleeding, such as melena or hematemesis, with an associated decrease in hemoglobin of at least 2 g/dL, or the need for blood transfusion.

### Clinical data

The collected data included information on demographic variables (sex, age), duration of ICU stay, outcome variables, time of ERCP, alkaline phosphatase (ALP), gamma-glutamyl transpeptidase (GGT), total bilirubin, ERCP findings, findings from other liver imaging studies, and blood and bile culture microbiology findings. The onset of cholestasis was defined according to the recommendations of the European Association for the Study of the Liver as elevation ALP >1.5 times the upper limit of normal (ULN) and elevation of GGT >3× ULN (14). Clinical and laboratory findings, baseline and follow-up enhanced abdominal computed tomography (CT), ultrasonography, endoscopic ultrasound (EUS), magnetic resonance imaging (MRI), and magnetic resonance cholangiopancreatography (MRCP) findings were extracted from the patient records. The diagnosis of liver abscess was established based on clinical, laboratory, and imaging (ultrasound, ERCP, and/or CT) findings. Abscess size was defined as the largest diameter of a single abscess or, in the case of multiple abscesses, as the largest diameter of the largest abscess. For electron microscopy, extracted biliary cast were fixed in 2.5% glutaraldehyde in cacodylate buffer (pH 7.3). Postfixation was performed with 1% OsO4 (osmium tetroxide) for 2 hours, followed by dehydration in an ascending ethanol series and finally critical point drying (CPD300; Leica Microsystems, Wetzlar, Germany). Finally, the samples were subjected to gold-palladium sputtering (Sputter Coater MED 020; Balzer, Bingen, Germany) and stored in vacuum until examination under a scanning electron microscope (GeminiSEM 300; Carl Zeiss, Oberkochen, Germany).

### Statistical analysis

Categorical variables were expressed as frequencies (n, %) and continuous variables as median with interquartile range (IQR) or mean ± SD. The Kaplan-Meier method was applied to calculate the probability of survival. The log-rank test was used to test for significant differences in survival among the subgroups. All statistical tests were 2-sided, and *P* values < 0.05 were considered statistically significant. Cox proportional hazard model was used to analyze the influence of several variables on LT-free survival in a multivariate approach. A multivariate analysis by means of logistic regression with stepwise selection (significance level for addition *P* < 0.05) was performed to identify prognostic factors. All statistical analyses were performed using Stata/IC 16.1 for Unix. The graphical abstract was created with BioRenter.com.

## RESULTS

Forty-six patients (12 female and 34 male) with a mean age of 57.0 ± 11.2 years were identified, and all were included in the study. Patient characteristics and procedures are summarized in Tables 1 and 2. Post–COVID-19 cholangiopathy occurred exclusively in critically ill patients with COVID-19 with invasive ventilation, but not in patients with noninvasive ventilation or mild COVID-19 (nonintensive care). Reverse transcription polymerase chain reaction–based analysis of bile samples from 8 patients with early ERCP showed no evidence of the presence of SARS-CoV-2 viral RNA.

**Table 1.**
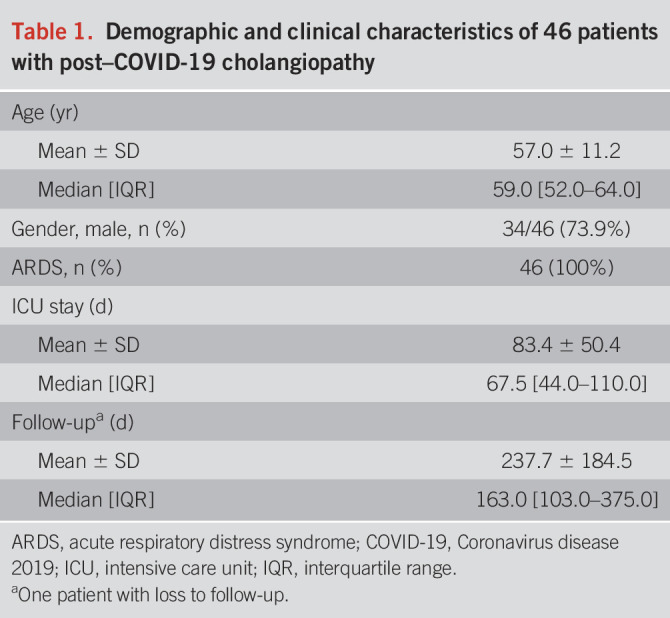
Demographic and clinical characteristics of 46 patients with post–COVID-19 cholangiopathy

**Table 2.**
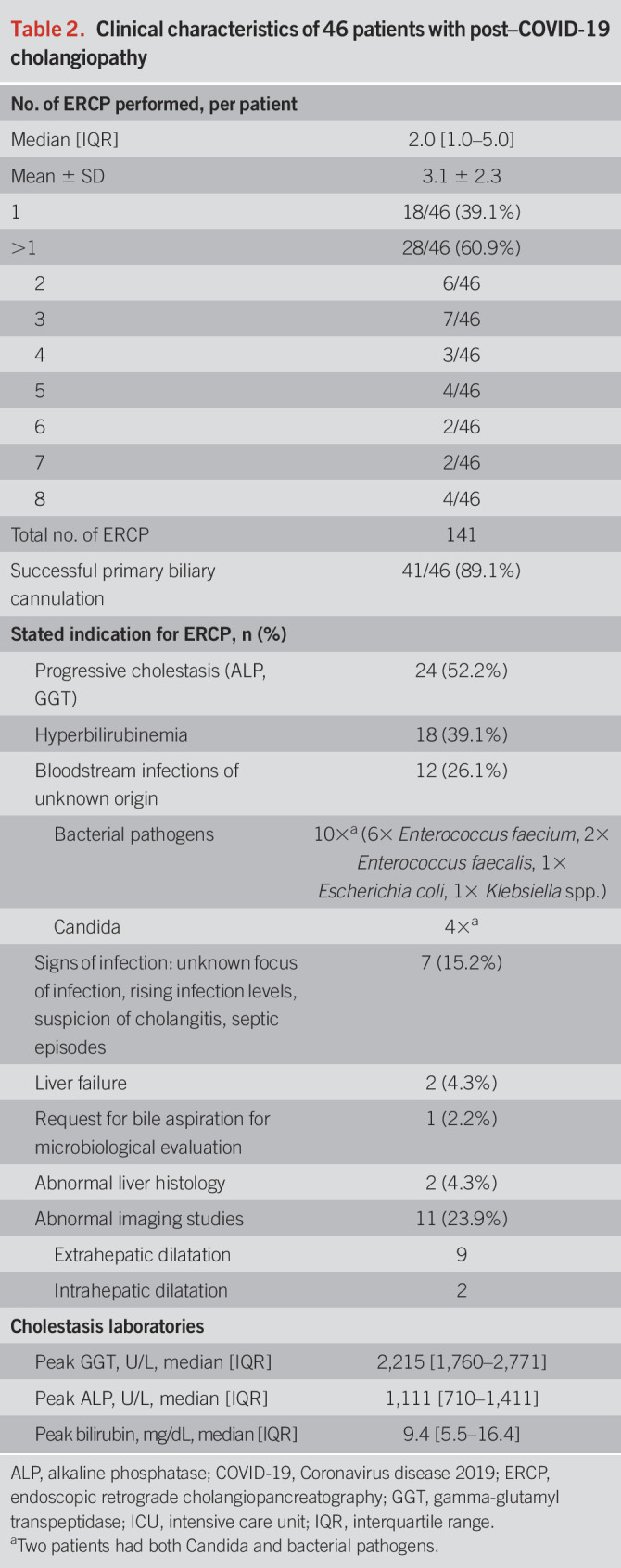
Clinical characteristics of 46 patients with post–COVID-19 cholangiopathy

Early onset of cholestasis was a characteristic feature of post–COVID-19 cholangiopathy. Cholestasis (defined according to the European Association for the Study of the Liver criteria) was first detected 4.5 days (median, IQR 3.0–7.0) after intubation. Cholestasis progressed from that day on and peaked on ICU days 25–36. GGT peaked on the 25th ICU day, ALP on the 32th ICU day, and bilirubin on the 36th ICU day (median, IQR GGT: 19–30; ALP: 26–39; bilirubin: 23–43). GGT increased to 36.3× ULN, ALP to 8.5× ULN, and bilirubin to 7.8× ULN (median, Table 2).

### Delay from onset of cholestasis to first ERCP

The time from cholestasis onset to the first ERCP was 50 days (median, IQR 34–81). Nine patients had their first ERCP within 30 days and 5 within 25 days of cholestasis onset. The need for ERCP most often arose during the ongoing ICU stay. Approximately 76.1% (35/46) of the patients were referred for ERCP from the ICU, 6 patients from the regular ward, and 5/46 patients were readmitted for ERCP after hospital discharge. In these readmitted patients, the ERCP was delayed until day 196 after the onset of cholestasis (median IQR 164–458, *P* < 0.001). A total of 141 ERCP were performed. Twenty-eight patients underwent multiple consecutive ERCP (Table 2). Initial biliary cannulation was successful in 89.1% of cases. Selective biliary cannulation failed in 5 patients but was possible in a second session. Stated reasons for ERCP referral are summarized in Table 2.

### Cholestasis levels before the first ERCP

All patients had relevant preinterventional cholestasis (1–2 days before ERCP) as evidenced by a median GGT of 1,532 U/L (IQR 1,178–2,011) and an ALP of 880 U/L (IQR 540–1,106), corresponding to 25.1× ULN (GGT<61 U/L) and 6.8× ULN (ALP <130 U/L), respectively. Total bilirubin was 9.8 mg/dL (median IQR 2.7–16.6) corresponding to 8.1× ULN (<1.2 mg/dL). However, up to 25.6% of patients had normal serum bilirubin despite documented cholangiographic abnormalities.

### Results of pre- ERCP imaging studies

Before ERCP, all 46 patients were examined using the following noninvasive imaging modalities: CT in 14/46, ultrasonography in 11/46, MRI/MRCP in 5/46, EUS in 4/46, CT + ultrasonography in 5/46, MRI + ultrasonography in 2/46, CT + MRI in 2/46, CT + MRI + ultrasonography in 1/46, and CT + EUS + MRI in 2/46. Extrahepatic dilatation was detected in 8/46 cases (17.4%), intrahepatic dilatation in 3/46 (6.5%), and combined intrahepatic and extrahepatic abnormalities in 1/46. Pre-ERCP imaging studies showed no dilatation in 74% (34/46) of cases. A negative imaging result delayed ERCP and the final diagnosis of post–COVID-19 cholangiopathy by 23 days (median, *P* = 0.057).

### Bile duct abnormalities detected by ERCP

#### Destruction of the intrahepatic bile ducts.

**Figure 1.**
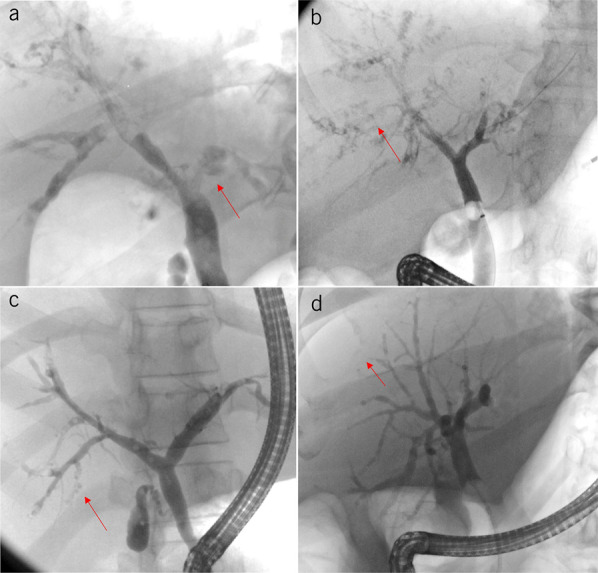
Distribution pattern of the biliary lesions. (**a**) Hepatic duct type: ERCP in a 60-year-old man with post–COVID-19 cholangiopathy shows involvement of the left hepatic duct and the subsegmental and segmental branches; the patient did not survive. (**b**) Segmental type: ERCP in a 59-year-old woman with post–COVID-19 cholangiopathy shows that both hepatic ducts are intact but the peripheral, subsegmental, and segmental branches are affected; the patient did not survive. (**c**) Subsegmental type: ERCP in a 48-year-old man shows that the peripheral and subsegmental branches are affected, while the segmental branches and both hepatic bile ducts are preserved; the patient was alive at the end of follow-up. (**d**) Peripheral type: ERCP in a 53-year-old woman shows that the peripheral branches are affected, while the subsegmental and segmental branches and both hepatic bile ducts are preserved; the patient survived. COVID-19, coronavirus disease 2019; ERCP, endoscopic retrograde cholangiopancreatography.

**Figure 2.**
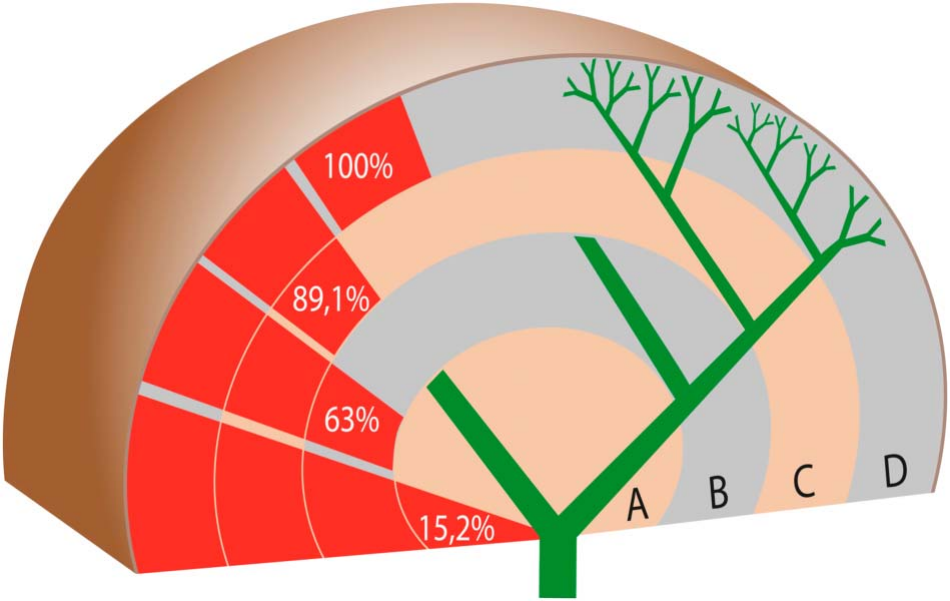
Anatomical distribution of biliary lesions in post–COVID-19 cholangiopathy. Frequency of each type of destruction in the overall cohort. (©2023/Christine Voigts/Charité.)

The cholangiographic findings of 141 ERCP studies were categorized. Based on the anatomical distribution of biliary lesions along the biliary tree (adapted from Buis) (15), the following 4 types of bile duct destruction (Figures 1 and 2) were distinguished:

1. Hepatic duct type: destruction of peripheral intrahepatic + subsegmental + segmental branches and right and/or left hepatic duct (type A)
2. Segmental type: destruction of peripheral intrahepatic + subsegmental + segmental branches (type B)
3. Subsegmental type: destruction of peripheral intrahepatic + subsegmental branches (type C)
4. Peripheral type: destruction of peripheral intrahepatic bile ducts alone (type D)

**Figure 3.**
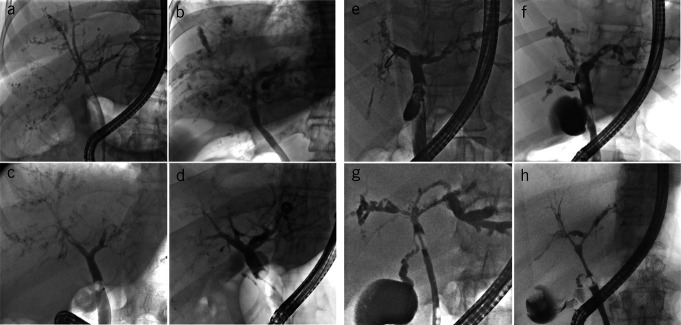
Wall destruction characteristic of post–COVID-19 cholangiopathy. (**a**–**d**) Early stages: ERCP in 4 patients with post–COVID-19 cholangiopathy showed destruction of peripheral, subsegmental, and (predominantly) segmental branches of the biliary tree. (**e**–**h**) Late stages in 4 different patients: advanced loss of intrahepatic branches. COVID-19, coronavirus disease 2019; ERCP, endoscopic retrograde cholangiopancreatography.

Peripheral bile ducts (type D) were affected in all 46 patients, an alteration up to the level of the subsegmental branches was detectable in 89.1%, and to the level of the segmental branches in 63% of cases. A destruction of the right and/or left hepatic duct was less common (15.2%). Patients with central destruction tended to have worse survival than those with peripheral destruction alone; the hazard ratio was 1.7 (CI 0.27–10.74), but the difference was not statistically significant (*P* = 0.52). Preferential destruction of the right intrahepatic bile ducts was observed in 56.5% of patients. All biliary lesions (100%) were intrahepatic, and no exclusively extrahepatic involvement occurred. Isolated biliary strictures near the hilum without concomitant destruction of upstream peripheral branches were not observed. Destruction of subsegmental branches was detected in some patients as early as 15 days after the onset of cholestasis. Destruction of bile duct wall was more prominent than the formation of circumscribed strictures (Figure 3). The common hepatic duct and the common bile duct (CBD) were spared from wall destruction in all 46 cases (Supplementary Digital Content, see Figure S1, http://links.lww.com/AJG/D91). ERCP revealed slight dilatation of the CBD (diameter ≤ 9 mm) in 8 patients and moderate dilatation (diameter 11 mm) in 1 case.

#### Phenomenon of vanishing bile ducts.

A dynamic change in the cholangiographic findings was observed in patients with follow-up ERCP. While early ERCP demonstrated only alteration of peripheral branches (type D), subsequent ERCP showed the gradual disappearance of subsegmental and segmental branches. Progressive centripetal “vanishing of bile ducts” was observed in 88.9% of the patients with the necessity for follow-up ERCP (Supplementary Digital Content, see Figure S2, http://links.lww.com/AJG/D92). The time to disappearance of 1 level of the biliary tree was 38 days (median, IQR 26–56).

#### Detection of biliary casts.

**Figure 4.**
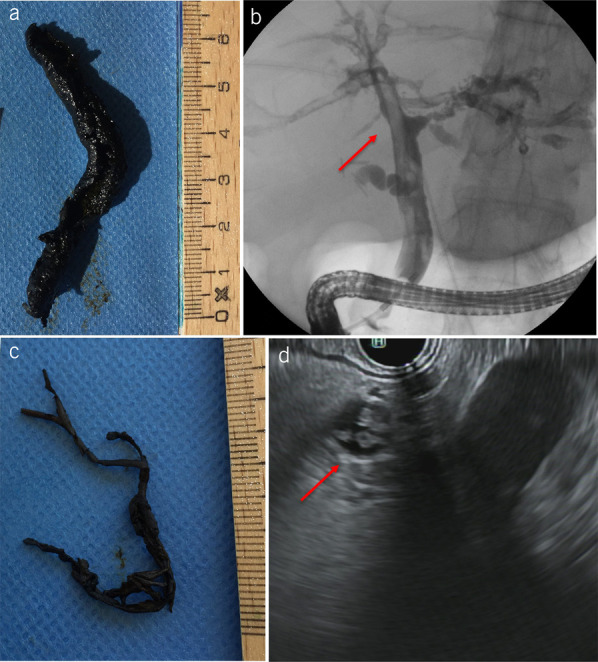
Biliary casts I. (**a** and **c**) Extracted casts of different shapes. (**b**) endoscopic retrograde cholangiopancreatography shows biliary casts as ribbon-like intraductal filling defects (arrow) (**d**) Radial endoscopic ultrasound (EUS) of the common bile duct shows biliary casts as hyperechogenic material (arrow). (**c** has been adapted from Leonhardt et al. doi: 10.1007/s00134-023-07257-8.)

**Figure 5.**
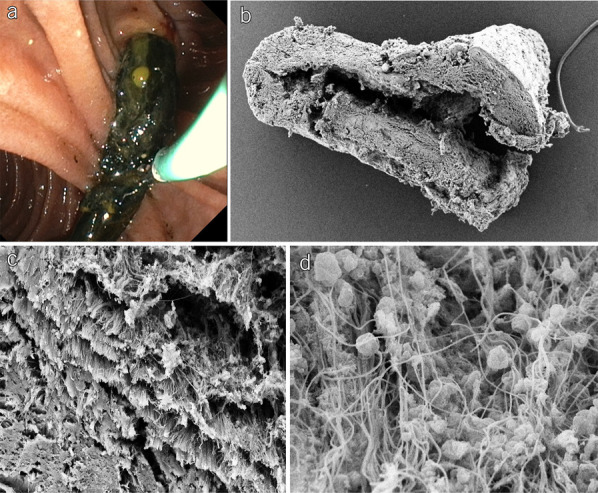
Biliary casts II. (**a**) Cast removal by endoscopic retrograde cholangiopancreatography. (**b**) Scanning electron microscopy of an extracted biliary cast from a 53-year-old woman with post–COVID-19 cholangiopathy (**c**) Scanning electron microscopy showed that the biliary casts contain collagen fibers. (**d**) Scanning electron microscopy showed casts overgrown by bacterial cocci.

Biliary casts were detected by ERCP in 39/46 patients (85%). The earliest detection of a cast was at day 15 and the latest at day 528 after the onset of cholestasis. ERCP revealed casts as elongated band-shaped filling defects of various sizes in the subsegmental branches, segmental branches, or the CBD. Casts that extended to the CBD were also identifiable by EUS in 4 of 6 patients (Figures 4 and 5). Cast-like material in the CBD was detected by CT in 1/24 patients and existing casts by MRI/MRCP in 2 of 11 patients.

#### Liver abscesses.

**Figure 6.**
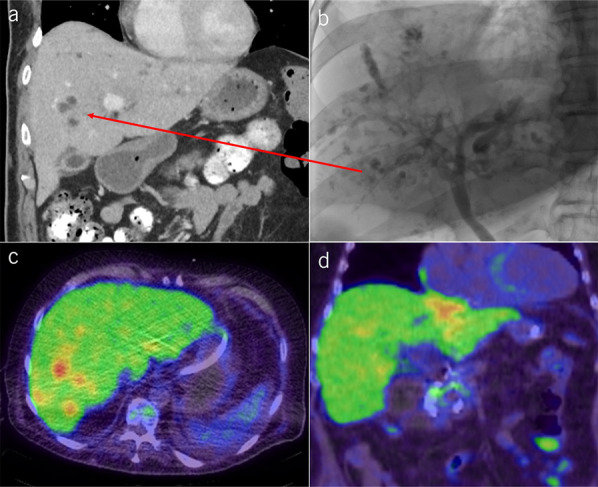
Liver abscesses in post–COVID-19 cholangiopathy. (**a**) CT of a 52-year-old man confirmed abscesses that were suspected in the ERCP 1 day before. (**b**) Same patient as in A: ERCP showed multiple small abscesses in the right hepatic lobe, the patient required LT. (**c**) Axial positron emission tomography-computed tomography showed multiple liver abscesses in a 60-year-old man with post–COVID-19 cholangiopathy. (**d**) Same patient as in C: frontal plane showed an abscess in the left liver lobe extending to the left ventricle. The patient did not survive. COVID-19, coronavirus disease 2019.

Fifteen patients (32.6%) had liver abscesses: in 40% in the right hepatic lobe alone and 60% had a bilateral involvement. Multifocal microabscesses (mean maximum diameter of 4.4 mm) were identified by ERCP in 12/15 patients, which mainly appeared as small round clusters of contrast medium along the bile ducts (Figure 6b). 7/15 patients had larger liver abscesses also situated along the bile ducts, which had a median maximum diameter of 20 mm (IQR 15.0–31.0). These larger abscesses were also detectable by other imaging techniques (CT, PET-CT, and MRI) (Figure 6). In all 15 patients with liver abscesses, multiple blood cultures had been obtained during the ICU stay, and 14 patients had bile cultures. The most commonly identified pathogens in bile cultures were *Enterococcus faecium* (12/14) and *Candida* spp*.* (12/14), followed by *Klebsiella* spp*.* (4/14). The median LT-free survival was 173 days in patients with peribiliary abscesses compared with 380 days in those without abscesses, but the difference was not significant. Patients with liver abscesses had worse survival rates than those of patients without abscess (31% vs 52% at 12 months; n.s.) (Supplementary Digital Content, see Figure S3, http://links.lww.com/AJG/D93).

#### Ductographic signs of post–COVID-19 cholangiopathy.

Ductography revealed the following changes: multiple ribbon-like intraductal filling defects (biliary casts), weak and sometimes indistinct (shadowy) contrast enhancement of the bile ducts, spotty and irregular contrast distribution within the ducts, long-distance irregularities of duct contour, lacunar contrast penetration/cavitation toward the parenchyma, biloma formation, and irregular narrowing of the bile ducts (Figure 3).

### ERCP-related complications

Adverse events are summarized in Table 3. Overall bleeding complications were observed in 11/46 patients (23.9%). All of these patients required blood transfusion with a median of 6.0 (IQR 3.0–12.0) RBC units. 6/10 patients required endoscopic hemostasis, which was successfully performed in 5 cases and failed in 1 case; the latter patient required an angiographic intervention to achieve hemostasis. The 11th case of bleeding developed a severe hemobilia due to pronounced necrosis of the left hepatic duct. No other ERCP-related complications (such as pancreatitis, infection, or perforation) were observed.

**Table 3.**
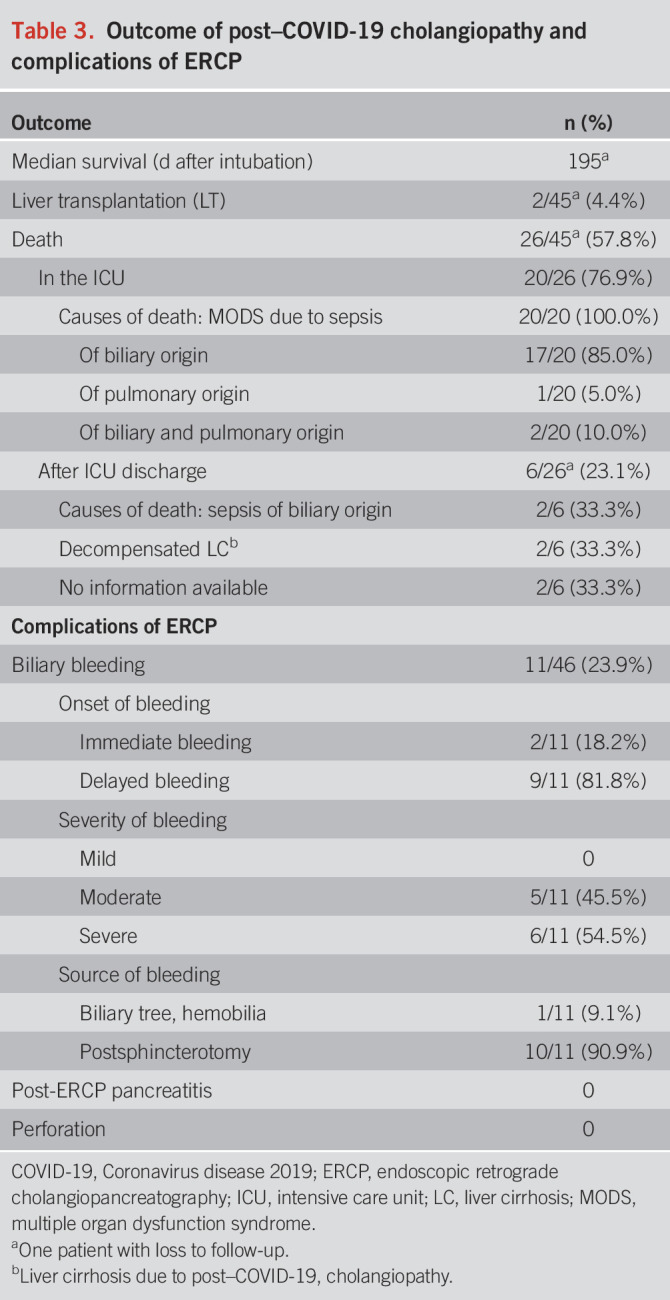
Outcome of post–COVID-19 cholangiopathy and complications of ERCP

### Performed endoscopic therapeutic interventions

Complete or partial cast removal by ERCP (with balloons, baskets, and forceps) was successfully conducted in 92.3% of patients with casts. The size of the removed casts ranged from a few millimeters to 9 cm in length. The larger casts took on the physical shape of the bile ducts or even imitated the complete bile duct tree (Figure 4). Casts were detected multiple times and removed on multiple occasions in 53% of patients with serial ERCP. Cholestasis parameters (i.e., GGT, ALP, and bilirubin) were recorded before and after cast extraction. Analysis of the recorded parameters revealed a decrease in 58.1% of all individual values. The GGT responded best to cast removal, 73.4% of GGT values declined while bilirubin improved in only 39.4% of the cases. Cholestasis parameters decreased by 22.1% (median) (IQR 8.7–39.3) compared with the pre-ERC baseline value within 10 days after ERC. Endoscopic stricture therapy was less common: only 6 patients (13%) had to be treated for central biliary tract strictures.

### Outcome of patients with post–COVID-19 cholangiopathy

**Figure 7.**
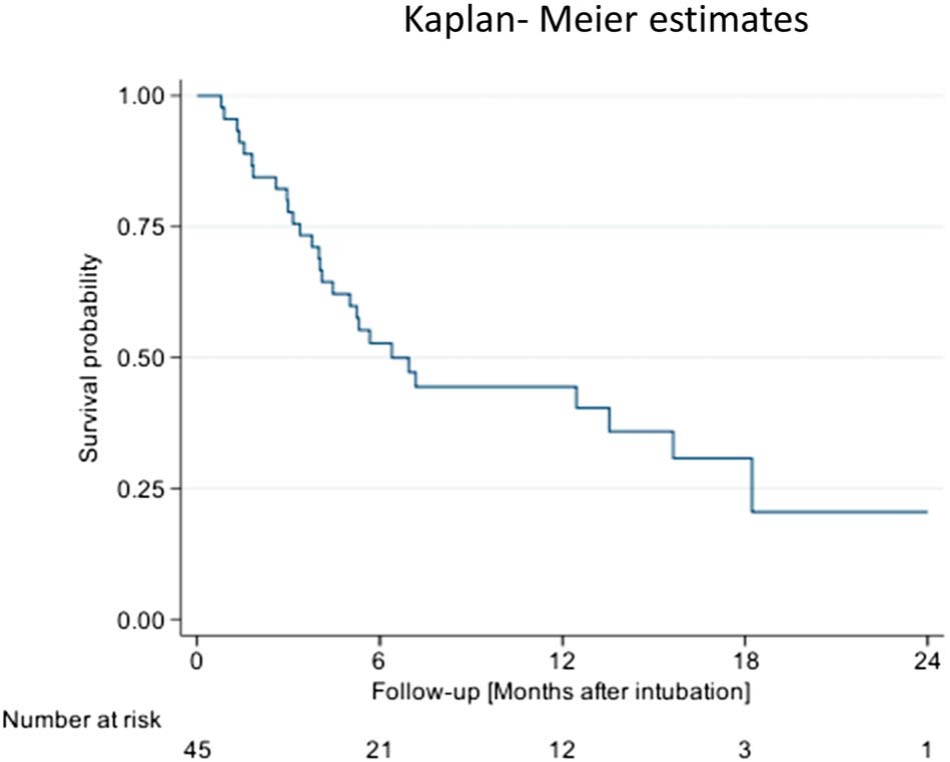
Kaplan-Meier survival curve for overall survival. A total of 45 patients with post–COVID-19 cholangiopathy were included in the analysis. One patient of the cohort who was lost to follow-up was excluded.

Survival rates for the cohort are shown in Figure 7. Early mortality was rather high: 20 of our patients (43.5%) died during their initial ICU stay. Two patients required LT, one of them 162 and the other 380 days after intubation for COVID-19. Six patients died after discharge, 17/45 have survived (1 patient was lost to follow-up after discharge from ICU). Most of our patients died of liver-related causes (Table 3). The 1-year transplant-free survival rate was 44%. Multivariate analysis identified bilirubin as an independent factor for negative outcome: the higher the bilirubin value in the first peak (day 25–36 after intubation), the shorter the LT-free survival (hazard ratio 1.08, *P* < 0.001).

## DISCUSSION

Post–COVID-19 cholangiopathy is one of the most serious long-term gastrointestinal consequences of COVID-19. Because of the therapeutic implications (e.g., the need for LT), a correct diagnosis is essential. Bile duct imaging plays a pivotal role in the diagnosis of this cholangiopathy. As we have shown in this study, ERCP is not only able to visualize the unique features of post–COVID-19 cholangiopathy but also provides prognostic information and has therapeutic value. The superiority of ERCP over noninvasive imaging modalities such as MRCP lies in its ability to detect fine bile duct wall irregularities (necrosis) and soft intraluminal filling defects (casts) and its sensitivity in detecting biliary lesions, even in small peripheral bile ducts (11,16).

Sparing of the CBD, centripetal destruction of the intrahepatic bile ducts, and the phenomenon of vanishing bile ducts were the typical cholangiographic patterns of post–COVID-19 cholangiopathy. This cholangiopathy was more destructive than stricturing in nature and caused irreversible damage to the bile ducts. As observed in this study, the spectrum of cholangiograhic findings ranged from mild peripheral biliary alterations up to complete destruction of the intrahepatic bile ducts. Destruction of the peripheral intrahepatic bile ducts (type D) was detected in all patients in our cohort. As the extent of damage increased, bile duct destruction spread centripetally toward the subsegmental or segmental branches. The central bile ducts (both hepatic bile ducts) were affected in only 15.2% of cases. Cholangiographically detectable biliary tract destruction gradually increased over time. Most patients with follow-up ERCP showed marked centripetal “vanishing” of the visible intrahepatic bile duct branches over time, resulting in a pruned tree appearance of the biliary tree, as was previously described for SSC-CIP (17). The vanishing phenomenon progressed very quickly. No other type of sclerosing cholangitis has ever caused such rapid obliteration of the bile ducts (18). Extensive ischemic necrosis and replacement with connective tissue could explain the rapid disappearance of the bile ducts (Supplementary Digital Content, see Figure S4, http://links.lww.com/AJG/D94). However, in some patients, the cholangiographic changes remained stable over time, highlighting differences in the degree of initial bile duct damage.

The presence of biliary casts, detected in 85% of our patients, was a prominent feature of post–COVID-19 cholangiopathy. Biliary cast syndrome (BCS) is a rare entity. First described by Waldram et al in 1975, BCS has been causally associated with only 2 etiologies. One is LT: biliary casts occur in 4%–18% of LT recipients (19–21). Outside the LT setting, BCS is only known to occur in association with SSC-CIP and is considered pathognomonic for this disease (17). The fact that BCS has never been seen in association with ketamine-induced or viral cholangiopathies in the past supports the classification of the post–COVID-19 cholangiopathy described here as a variant of SSC-CIP. Although the exact mechanism of cast formation is unclear, the association with LT and SSC-CIP suggests that ischemia plays a role. The presence of collagen fibers in the biliary casts, as was also demonstrated by electron microscopy in our series (Figure 5c), suggests that epithelial necrosis (with release of collagen fibers) is a prerequisite for cast formation (22). Pre-ERC noninvasive imaging could not reliably predict the presence of casts. In line with the results of a previous study, MRI/MRCP correctly diagnosed BCS in only 18.2% of our patients (23).

Peribiliary liver abscesses, found in more than one-third of our patients, were another characteristic feature of post–COVID-19 cholangiopathy. Due to their small size and multifocal appearance along the biliary tree, these microabscesses could not be treated by an interventional approach (e.g., percutaneous drainage). Therefore, targeted antibiotic therapies are all the more important in these cases. Usually, *Escherichia coli* and *Klebsiella pneumoniae* are the most common pathogens of liver abscesses, whereas enterococci are rather rare, with a prevalence of 4%–7% (24–27). In our cohort, however, enterococci (*E. faecium*: 86%) and *Candida* spp. (86%) were the predominant pathogens in bile. This emphasizes the importance of microbiological examination for pathogen-specific therapy, as possible by an ERCP with bile sampling. Generally, liver abscesses are still associated with significant mortality, with reported rates ranging from 6% to 10% (28–31). This is also reflected in our data: the presence of abscesses worsened LT-free survival of our patients.

Our patients with post–COVID-19 cholangiopathy had a poor prognosis. Almost half of them died during their initial stay in ICU. In our cohort, the 1-year LT-free survival was only 44%, which is even worse than the recently reported survival rates for SSC-CIP in general (32). We also evaluated the impact of ERCP cholangiographic findings on survival. The 2 ERCP findings that appear to be of prognostic importance are the presence of peribiliary abscesses and the extent of bile duct damage. Our patients with liver abscesses tended to have poorer survival. Our cholangiographic classification system divides the severity of post–COVID-19 cholangiopathy into 4 types (A-D), reflecting the extent of bile duct damage. The finding that patients with hilar involvement (type A) tend to have worse survival than those with only peripheral involvement suggests that patients with signs of advanced bile duct destruction in ERCP require closer clinical management.

ERCP also has therapeutic value. Cast removal to improve biliary drainage was frequently performed by ERCP in our study. Previous SSC-CIP studies have reported biochemical improvements after cast extraction (33), but our results only partially confirmed this because only approximately 60% of all recorded individual cholestasis parameters improved. In a small proportion of our patients, cholestasis parameters did not decrease or even increased. The cholestasis level in post–COVID-19 cholangiopathy is most likely multifactorial. In addition to cast-related obstruction, necrosis of the bile duct epithelium and liver abscesses probably also play a role. Thus, unlike stone removal in choledocholithiasis, cast removal does not always lead to a measureable improvement of laboratory parameters. Nevertheless, we hypothesize that biliary casts should be removed because they contribute to biliary obstruction, provide a nidus for pathogens, and may promote secondary bacterial inflammation (Figure 5d). In our study, biliary casts remained detectable for 18 months. Within this time, ERCP to remove casts may be useful. Another endoscopic procedure performed by ERCP was endoscopic balloon dilatation of bile duct strictures. Bölter et al (34) reported that consistent stricture therapy extended transplant-free survival from 8.6 to 89 months in SSC-CIP patients. Unfortunately, we could not confirm such a positive effect in our cohort. Pronounced necrotic bile duct destruction made endoscopic therapeutic intervention impossible in some cases. When secondary strictures were present, they were predominantly located in the smaller intrahepatic branches beyond (upstream of) the left and right hepatic ducts and were not suitable for endoscopic dilatation. Overall, only 13% of our patients with central strictures underwent endoscopic balloon dilatation.

Our data draw attention to an issue regarding the diagnostic workup of “cholestasis” in critically ill patients. In our study, ERCP referrals most commonly originated from the intensive care setting, predominantly for clarification of progressive cholestasis (Table 1). Cholestasis is common in ICU patients, requires careful differential diagnosis, and remains a major challenge (14,35,36). Noninvasive imaging is recommended as the initial workup to evaluate the type of cholestasis (i.e., intrahepatic or extrahepatic cholestasis). ERCP should only be considered if there is evidence of bile duct dilatation (14,37). However, this diagnostic approach to cholestasis is not applicable to ICU patients with suspected post–COVID-19 cholangiopathy (and SSC-CIP in general). The minority of our patients (26%) had extrahepatic or intrahepatic bile duct dilatation in pre-ERCP imaging studies. Due to negative imaging, there was a delay in final diagnosis in patients without evidence of dilatation. Recommendations for the diagnostic workup of cholestasis in ICU patients should address this problem.

Of the known adverse events associated with ERCP, postendoscopic sphincterotomy bleeding (post-ESB) was the most common complication in this study. The rate of post-ESB was unusually high in our patients—almost 22% compared with only 1.3%–2% in unselected patient populations (38,39). ICU patients represent a particularly critical patient cohort. However, Buechter et al found no relevant increase in the rate of postsphincterotomy bleeding (2.7%) in ICU patients. Consistent with the literature, we predominantly observed delayed bleedings (40). Although the rates reported in other studies tend to be lower (25%–29%), post-ESB was severe in 50% of our cases (39,41). Known risk factors of postsphincterotomy bleeding, such as anticoagulant therapy, platelets <50,000/mm³, cirrhosis, low endoscopist experience, and end-stage renal disease, were not observed and do not explain our findings. The COVID-19–associated coagulopathy makes patients prone to bleeding. Recent studies have shown that critically ill patients with COVID-19 are at a particularly increased risk of gastrointestinal bleeding (42). The increased bleeding diathesis in our series may be related to the pathophysiology of COVID-19.

Previous studies have shown that GGT is the best laboratory indicator of the development of SSC-CIP (13,43). Our results confirmed this. In our cohort, cholestasis was mainly characterized by GGT elevation. GGT was the parameter that peaked first and increased the most (36× ULN). Conversely, total bilirubin cannot be used to predict the development of post–COVID-19 cholangiopathy because one quarter of our patients had normal bilirubin levels. Nevertheless, our multivariate analysis showed that bilirubin is a suitable parameter for estimating prognosis: the greater the bilirubin increase between ICU days 25 and 36, the worse the outcome.

Our data provide some insights into the pathophysiology of post–COVID-19 cholangiopathy. The delayed diagnosis of post–COVID-19 cholangiopathy may lead to the misinterpretation that this entity requires a long-term stay in the ICU (10). The early onset of cholestasis, demonstrated in our study, contradicts this view and suggests that the damage to the bile ducts is an early event. It most likely occurs when the patient's condition is most critical, i.e., around the time of intubation. Both the peracute onset of post–COVID-19 cholangiopathy and the distribution pattern of bile duct lesions are reminiscent of ischemic cholangitis and confirm our recent findings (9). PCR testing, although not performed in all of our patients, did not detect any viral components of SARS-COV-2 in the bile. Therefore, our results do not support the hypothesis of a viral pathogenesis of post–COVID-19 cholangiopathy.

Conclusion: As demonstrated by ERCP, the cholangiographic features of post–COVID-19 cholangiopathy suggest that it is a variant of SSC-CIP. ERCP is the method of choice for establishing the diagnosis and prognosis of post–COVID-19 cholangiopathy and has many therapeutic applications, such as bile sample collection for pathogen-specific therapy and biliary cast removal. Understanding the highly variable endoscopic findings of post–COVID-19 cholangiopathy is a prerequisite for adequate endoscopic management adapted to the course of the disease. The changes found in this study are typical of SSC-CIP in general.

## CONFLICTS OF INTEREST

**Guarantor of the article:** Silke Leonhardt, MD.

**Specific author contributions:** S.L. and C.J.: were responsible for the study conception and design. S.L., C.J., and H.J.J.: identified cases eligible for study inclusion. S.L., C.J., and D. Grajecki: collected the clinical data for the Charité patients. S.L. and C.J. had full access to all the study data and ensured the integrity of the data and the accuracy of the data analysis. H.J.J. and T.K.: collected the data for patients from Unfallkrankenhaus Berlin and anonymized the data for statistical analysis. S.L. and J.L.: analyzed the data, designed the figures, contributed to the statistical analysis, and wrote the first draft of the manuscript. S.L., C.J., A.A., D.Geisel, U.F., H.J.J., T.K., S.F., and F.T.: treated the patients. All authors read the manuscript and contributed to critical revision of the manuscript for important intellectual content.

**Financial support:** None to report.

**Potential competing interests:** None to report.

**Study Highlights**

**WHAT IS KNOWN**

✓ New-onset cholangiopathy may occur in association with coronavirus disease 2019 (COVID-19).
✓ Post–COVID-19 cholangiopathy presents with cholestasis; the exact pattern of cholestasis has not been described.
✓ A poor prognosis was suspected, and endoscopic features are insufficiently described.

**WHAT IS NEW**

✓ Endoscopic features of post–COVID-19 cholangiopathy are described in detail and diagnostic criteria are provided.
✓ Diagnostic criteria are as follows: destruction of intrahepatic bile ducts in a centripetal direction, no extrahepatic involvement, phenomenon of vanishing bile ducts, biliary cast syndrome, and multifocal peribiliary abscesses.
✓ Post–COVID-19 cholangiopathy is not a late complication of intensive care treatment; rather bile duct damage is an early event in the course of COVID-19.
✓ The prognosis is dismal, and the impact of morphological changes on the outcome are described.
